# NVP-BEZ-235 enhances radiosensitization via blockade of the PI3K/mTOR pathway in cisplatin-resistant non-small cell lung carcinoma

**DOI:** 10.18632/genesandcancer.27

**Published:** 2014-07

**Authors:** Kwang Woon Kim, Carey J. Myers, Dae Kwang Jung, Bo Lu

**Affiliations:** ^1^ Department of Pediatric Surgery, Vanderbilt University School of Medicine, Vanderbilt University Medical Center, Nashville, TN.; ^2^ Department of Hematology/Oncology, Vanderbilt University School of Medicine, Vanderbilt University Medical Center, Nashville, TN.; ^3^ Department of Radiation Oncology, Thomas Jefferson University, Philadelphia, PA.

**Keywords:** mTOR, NSCLC, autophagy, cisplatin resistance, radiosensitization

## Abstract

**Introduction:**

Most drug resistant cancer cells also develop resistance to radiation therapy. In this study, we hypothesized that the dual inhibitor of phosphatidylinositol-3 kinase/mammalian target of rapamycin, NVP-BEZ-235, could potentially enhance radiosensitization in cisplatin-resistance (CDDP-R) non-small cell lung cancer (NSCLC) cells by disabling autophagy as a mechanism of self-preservation.

**Methods:**

We used both *in vitro* and *in vivo* approaches, including clonogenic assays, Western blotting, molecular analyses of autophagy and apoptosis, a xenograft model of tumor growth, and immunohistochemical analysis.

**Results:**

Basal p-Akt, p-mTOR and p-S6R proteins were enhanced in CDDP-R NSCLC cells. CDDP-R-resistant NSCLC cells are less radiation sensitive in comparison to parental cells (DER=0.82, p=0.02); BEZ-235 enhanced the radiosensitivity (DER=1.2, p=0.01). In addition, combining BEZ-235/RT showed a dramatic tumor growth delay in a mouse xenograft model. Immunohistochemistry showed that combination therapy yielded 50% decrease in caspase-3 activity. Moreover, cell proliferation was reduced by 87.8% and vascular density by 86.1%. These results were associated with a downregulation of PI3K/mTOR signaling pathway and an increase in autophagy.

**Conclusions:**

These findings may be utilized as a novel strategy to enhance the efficacy of radiation therapy in drug-selected non-small cell lung cancer exhibiting radioresistance.

## INTRODUCTION

Lung cancer is the leading cause of cancer-related mortality in the United States and the world [1-2]. Nearly 85% of these are non-small cell lung carcinoma (NSCLC), which typically has a poor prognosis; at 5 years post-diagnosis, survival rate is ~15%. One of the continuing challenges in the therapeutic approach towards NSCLC is the development of resistance to chemotherapy and radiation treatment. As a result, there has been a significant amount of research aimed at the identification of novel agents capable of overcoming these adaptations. Several of these agents are designed to target the PI3K pathway, as deregulation and aberrant activation of PI3K-α and its downstream targets have been linked to tumorigenesis and tumor maintenance in a variety of cancers, including lung, breast, prostate, colon, and brain cancers [3].

The PI3K pathway is implicated in many disparate cellular functions, including cell growth, proliferation, inhibition of apoptosis, metabolism, survival, and intracellular trafficking [4]. Many of these functions occur as a result of PI3K activating its downstream effectors Akt and mammalian target of rapamycin (mTOR), a kinase that participates in the regulation of cell growth, angiogenesis, and amino acid and glucose metabolism [5]. Deregulation can result from mutations or enhanced expression of the PI3K-α gene (PIKC3A) [6-7], activation of Ras or associated receptor tyrosine kinases [8], or loss of phosphatase and tensin homolog (PTEN, a negative regulator of PI3K signaling). Both PTEN loss [9] and PIKC3A gene copy amplification have been reported in NSCLC [10-11]. Irrespective of the mechanism, the frequent enhancement of PI3K/Akt/mTOR activation suggests in lung cancer suggests an important role for the pathway in carcinogenesis, thus providing strong impetus for the development of targeted agents directed at inhibiting the PI3K/Akt/mTOR pathway. Such inhibition may increase sensitivity to traditional chemo- and/or radiotherapy. One of the mechanisms by which this sensitization may occur is via autophagy.

Autophagy, the phenomenon of cellular self-digestion, has the potential to promote either cell death or cell survival in the context of different cancers [12-14] and post-irradiation [13, 15]. At baseline, it contributes to the health of the cell by recycling non-useful compounds, including damaged organelles and cytotoxic aggregates, and can be upregulated in response to cellular stress to combat infection or increase survival in suboptimal growth conditions [12-14]. Conversely, it can also contribute to type II (autophagic) cell death [12, 14]. The difference between these outcomes may be determined by the molecules and pathways activated during this process, which include Akt-mTOR^21^. Research is currently focused on weighting the autophagocytic response towards death, which has the added benefit of increasing tumor radiosensitivity [13, 16, 17]. Inhibiting autophagy in osteosarcomas increases sensitivity to chemotherapy [12]; concurrent radiation and autophagy inhibition increases DNA ds breaks in glioma cells [15] and increases cell death in the A549 lung cancer model [12]. Autophagy inhibitors such as 3-MA (a class-III-PI3K inhibitor), bafilomycin A1, hydroxychloroquine and monensin (autophagosome-lysosome fusion inhibitors) have shown different efficacies in mitigating the effects of chemo- and radiotherapeutic anti-cancer treatments [14], indicating the complexity with which this process is executed.

Recently, several new classes of drugs targeting the PI3K/Akt/mTOR pathway have been developed to combat the problems of earlier agents, including unfavorable pharmacokinetics, toxicity, and poor selectivity [18]. NVP-BEZ-235 is a synthetic imidazo[4,5-c]quinoline derivative compound that acts as a selective dual pan-class I PI3K and mTOR kinase inhibitor [19], and reversibly binds the ATP-binding sites of class I PI3K and mTOR kinase, inhibiting their catalytic activities; it has been shown to inhibit PI3K/Akt/mTOR signaling and has anti-proliferative and anti-tumor activity in several cancers, including breast cancer [20], glioma [21], lymphoma [22], NSCLC [23], pancreatic cancer [24], and renal cell carcinoma [25]. In this study, we investigate the role of the PI3K/Akt/mTOR signaling pathway inhibition via BEZ-235 in the radiosensitization of a cisplatin-resistant (CDDP-R) NSCLC cell line. Our results suggest that there is value in utilizing BEZ-235 to sensitize CDDP-R NSCLC to radiation, and this effect appears to be caused by increases in autophagy.

## RESULTS

### CDDP-resistant H460-Luc2 cells resemble lung cancer stem cells

**Figure 1:**
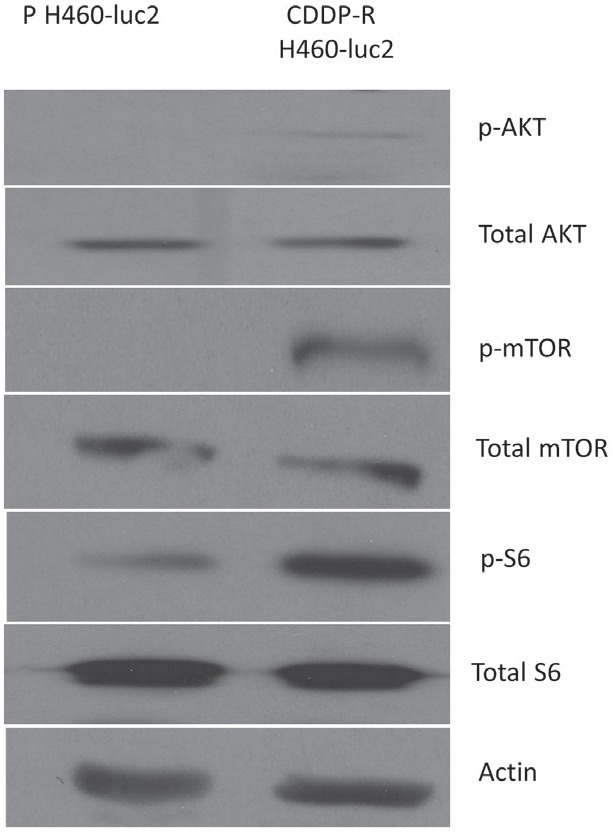
Cisplatin-resistant H460-luc2 cells show an increased expression of stem cell markers and increased activity of the AKT-mTOR pathway Cisplatin resistant (CDDP-R) H460-luc2 cells show an increase in the phosphorylation of AKT, mTOR, and the S6 ribosomal subunit, without a change in total protein expression.

H460-Luc2 lung cancer cells were selected for CDDP-resistance as described in the materials and methods. Briefly, NCI-H460-Luc2 cells were treated with 3μM cisplatin, and resistant cells were selected and expanded via growth in 1% methylcellulose-based media. To determine whether the generated CDDP-R cells exhibited certain stem cell-like features, cell lysates of parental and CDDP-R H460-Luc2 were subjected to Western blot analysis. Immunoblotting showed that CDDP-R H460-Luc2 had increased phosphorylation of AKT (Ser 473), mTOR (Ser 2448), and the S6 ribosomal subunit (Ser 240/244), without a change in total protein expression (Figure 1). These results suggest that that CDDP-R exhibits features of stem-like cells via activation of AKT/mTOR/S6 ribosomal protein axis.

### BEZ-235 treatment sensitizes CDDP-R H460-luc2 cells to cisplatin via inhibition of the PI3K/Akt/mTOR pathway

**Figure 2:**
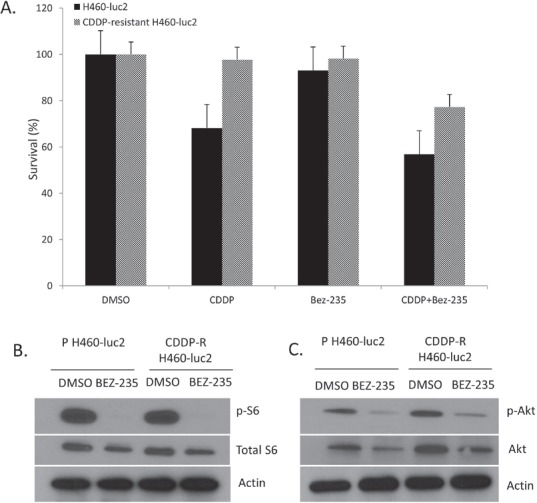
BEZ-235 treatment sensitizes CDDP-R H460-luc2 cells to cisplatin via inhibition of the PI3K/Akt/mTOR pathway Treatment of cisplatin-resistant H460-luc2 cells with concurrent BEZ-235 and cisplatin decreases cell survival. Cells were treated with DMSO (control), 3μM cisplatin, 50nm BEZ-235, or both (A). The parental cell line was susceptible to cisplatin treatment; neither cell line was significantly affected by BEZ-235 alone. Combining cisplatin and BEZ-235 reduced survival in both the parental and cisplatin-resistant cell lines. Treatment with 50nM BEZ-235 eliminates ribosomal S6 phosphorylation in both cisplatin-resistant H460-luc2 cells and the parental cell line (B). Both total and phosphorylated AKT were decreased in both cell lines, but the effect was more pronounced in cisplatin-resistant cells (C).

To determine whether BEZ235 is capable of restoring chemosensitivity in cisplatin-resistant cells, the CDDP-R and parental H460-Luc2 cell lines were treated with DMSO, 3μM cisplatin, 50nm BEZ-235, or combined cisplatin and BEZ-235. Analysis of an MTS (proliferation) assay demonstrated that CDDP-R cells were resistant to 3μM cisplatin treatment, while the parental cell line showed decreased survival (Figure 2A). Treatment with 50nm BEZ-235 alone showed no significant effect on survival of either parental H460-Luc2 or CDDP-R H460-Luc2 cell lines. Though combination treatment of the parental cell line showed significantly decreased survival from that after BEZ-235 alone (p=0.026), this was not seen when comparing survival after combination treatment to that after cisplatin alone (p=0.088). Similarly, combination treatment was significantly better than BEZ-235 treatment (p=0.018) but not cisplatin treatment in decreasing survival of CDDP-resistant cells (p=0.08). Treatment with 50nM BEZ-235 eliminates ribosomal S6 phosphorylation in both cisplatin-resistant H460-luc2 cells and the parental cell line (2B). Both total and phosphorylated AKT were decreased in both cell lines, but the effect was more pronounced in cisplatin-resistant cells (2C). Treatment with BEZ-235 resensitizes CDDP-resistant H460-Luc2 cells to cisplatin treatment by decreasing activity of the PI3K/Akt/mTOR signaling pathway.

### BEZ-235 significantly enhances the radiosensitivity of CDDP-R H460-Luc2 and parental cells

**Figure 3:**
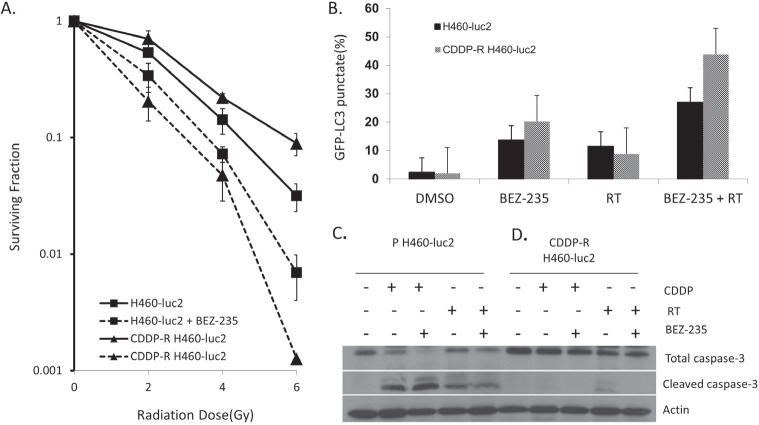
BEZ-235 significantly enhances the radiosensitivity of CDDP-R H460-Luc2 and parental cells via the induction of autophagy Parental or cisplatin-resistant cells were pretreated with 50nM BEZ-235 or DMSO for 24 hours, followed by irradiation with 0, 2, 4 or 6Gy (A). Untreated CDDP-resistant cells show greater resistance to radiation than the parental cell line (p=0.006). Treatment with BEZ-235 greatly increases radiation sensitivity in both the parental and CDDP-resistant cell lines (p=0.005 and p=0.007); this effect is greater in the radiation-resistant cell line (DER=1.5 vs 1.8). The difference in survival is much higher for the CDDP-resistant cells (p=0.007). Treatment with 50nm BEZ-235 for 2 hours and subsequent administration of 5Gy radiation induces autophagy in CDDP-R H460-Luc2 cells (B). Both parental and radiation-resistant cell lines treated with either BEZ-235 or radiation had increased levels of autophagy; treatment with BEZ-235 was more effective in both cell lines. Combined radiation and BEZ-235 treatment resulted in a significant enhancement in the levels of autophagy in both cell lines. In the parental cell line, this effect was additive; in CDDP-R cells these treatments acted synergistically, resulting in a significant increase in measurable levels of autophagy. Parental H460-Luc2 cells exhibited increased cleaved caspase-3 expression following treatment with RT, BEZ-235 + RT, 3μM cisplatin, or cisplatin + RT (C). The highest increased in expression was seen in cells treated with BEZ-235 and cisplatin; the cisplatin alone group showed the next highest level. BEZ-235 + RT induced less cleaved caspase-3 than RT alone. In CDDP-R cells, RT alone resulted caspase-3 cleavage (D). Co-treatment with BEZ-235 and RT, BEZ-235 and cisplatin, or treatment with cisplatin alone did not result in any measurable apoptosis, as determined by cleaved-caspase 3 expression.

To elucidate the effect of the administration of BEZ-235 on the sensitivity of CDDP-R H460-Luc2 and parental H460-Luc cell lines to radiation, cells were pretreated with 50nM BEZ-235 or DMSO for 24 hours, followed by irradiation with 0-6Gy. *In vitro* clonogenic assays demonstrate that CDDP-R H460-Luc2 cells are less sensitive to radiation when compared to parental cells (p=0.006) (Figure 3A). BEZ-235 significantly enhanced the radiosensitivity of both CDDP-R cells (DER=1.5) and parental cells (DER=1.8). Notably, BEZ-235 treatment resulted in significantly greater radiation sensitization of CDDP-R cells relative to parental cells (p=0.007). The radiosensitization effect of BEZ-235 is more pronounced in CDDP-resistant cells.

### Enhanced radiosensitivity of CDDP-R NSCLC is associated with an increase in autophagy; increased chemosensitivity is not linked to apoptosis

To further investigate the mechanism of enhanced killing in CDDP-R H460-Luc2 cells, both CDDP-R and parental cell lines were transfected with 2.5μg of GFP-LC3 expression plasmid, followed by treatment with 50nm BEZ-235 for 2 hours and subsequent administration of 5Gy radiation. Confocal microscopy analysis showed that both parental and radiation-resistant cell lines treated with either BEZ-235 or radiation had increased levels of autophagy; treatment with BEZ-235 alone was more effective (Figure 3B). Combined radiation and BEZ-235 treatment resulted in a significant enhancement in the levels of autophagy in both cell lines. In the parental cell line, this effect was additive (vs BEZ-235, p = 0.0006; vs RT, p=0.0004); in CDDP- R cells these treatments acted synergistically, resulting in a significant increase in measurable levels of autophagy (vs BEZ-235, p = 0.013; vs RT, p=0.0096) (Figure 3B).

To determine if the enhanced chemo and radiosensitivity demonstrated in Figures 2 and 3A are associated with increases in apoptosis, CDDP-R and parental H460-Luc2 cells were subjected to the following treatments: 50nM BEZ-235 or DMSO, followed by irradiation with 5Gy; cisplatin; or cisplatin followed by irradiation with 5Gy. 48h post-treatment, cell lysates were subjected to Western blot analysis and probed for cleaved caspase-3, a marker of apoptosis. Parental H460-Luc2 cells exhibited increased cleaved caspase-3 expression following all four treatments (Figure 3C). The highest increased in expression was seen in cells treated with both BEZ-235 and CDDP; the CDDP alone group showed the next highest level. Surprisingly, the combination of BEZ-235 and radiation induced less cleaved caspase-3 than RT alone. Notably, in CDDP-R cells, only treatment with radiation alone resulted in any measurable cleaved caspase-3 levels. As such, co-treatment with 50nm BEZ-235 and radiation did not result in any measurable apoptosis, as determined by cleaved-caspase 3 expression.

The enhanced radiosensitivity induced by BEZ-235 treatment is linked to an increase in autophagy; this effect is more pronounced in CDDP-resistant cells. The resensitization of CDDP-R cells to cisplatin is not linked to increased apoptosis, although radiation treatment alone does result in increased caspase-3 cleavage.

### Combining radiation and BEZ-235 delays growth of cisplatin-resistant tumors

**Figure 4:**
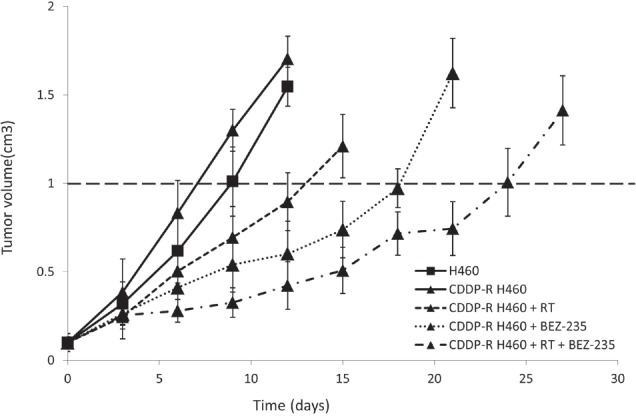
Treatment with BEZ-235 and RT delays tumor growth Tumors consisting of cisplatin-resistant cells grew more rapidly than tumors derived from the parental cell line when animals were untreated. BEZ-235 treatment delays tumor growth more than RT. Combination therapy, BEZ-235 + RT, significantly delays tumor growth.

Cisplatin-resistant tumors grow more quickly than those derived from the parental H460-luc2 cell line (Figure 4). Whereas tumors derived from CDDP-R H460 cells reached an average of 1cm^3^ after 7 days, those grown after the implantation of parental H460 cells required an extra 2 days (Figure 4). Although BEZ-235 treatment did not appreciably decrease cell growth in the cisplatin-resistant cell line *in vitro* (Figure 2), the *in vivo* model demonstrated a tumor growth delay of 6 days (Figure 4). However, this delay was not as great as that induced by radiation treatment (11 days). Consistent with the results shown in Figure 3, we found that combining BEZ-235 and RT greatly delays tumor development (Figure 4).

### RT, BEZ-235, and combination therapy alter expression of caspase-3, Ki67, p62, and von Willebrand factor in cisplatin-resistant cells

**Figure 5:**
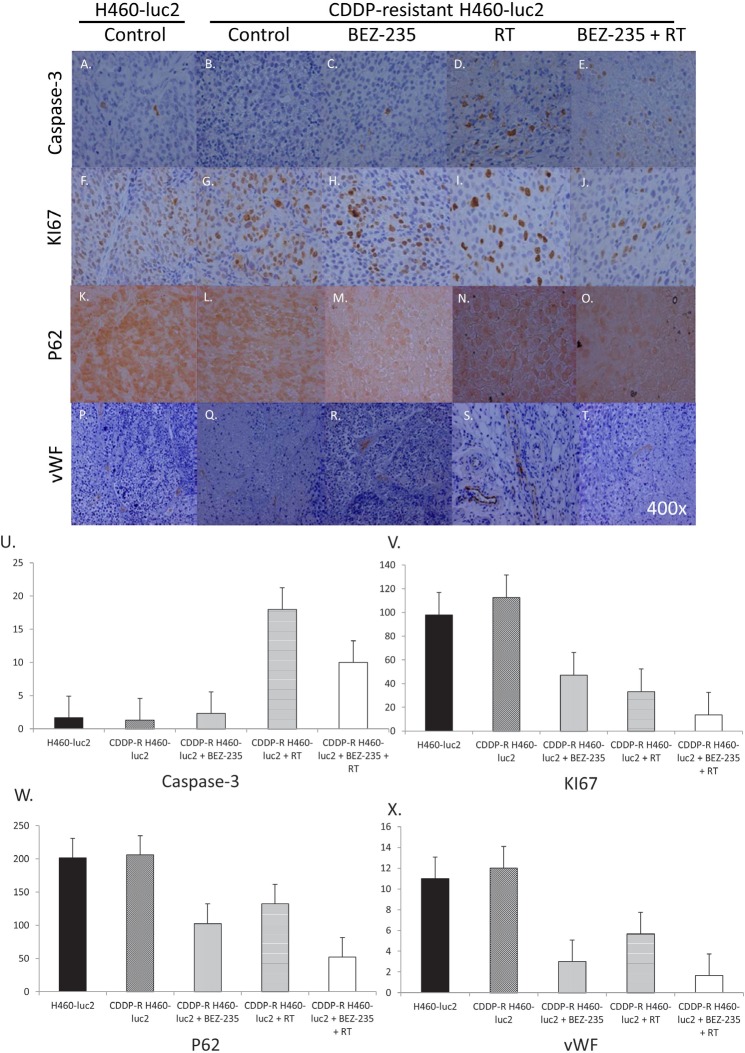
RT, BEZ-235, and combination therapy alter expression of caspase-3, KI67, p62, and von Willebrand factor in cisplatin-resistant cells Untreated CDDP-R cells produce slightly less caspase-3 than the parental cells (A, B; U). Caspase-3 is increased slightly after BEZ-235 treatment (C, U) and significantly after irradiation (D, U); combinatory treatment shows an intermediate effect (E, U). Cell proliferation (as measured by KI67 staining) was decreased by both BEZ-235 (58.2%; H, V) and RT (70.3%; I, V), and combination treatment (87.8%; J, V). Confirmation of this decrease was obtained after staining for p62. p62 expression is decreased by BEZ-235 (50%; M, W), RT (35.4%; N, W), and combination therapy (74.5%; O, W). Finally, angiogenic potential was assessed by staining for von Willebrand's factor. CDDP-R cells produce slightly more vWF than the parental cells at baseline (Figure 6P, Q). BEZ-235 decreases vWF expression by 75% (R, X), whereas RT decreases vWF expression in CDDP-R cells 48% (S, X). Combining radiation and BEZ-235 decreases vWF expression 86.1% (T, X).

In order to assess the effect of BEZ-235 on apoptosis, cells were treated with BEZ-235, RT, or a combination of the two and the production of several proliferation-related proteins assessed via IHC (Figure 5). At baseline, CDDP-R cells produce slightly less caspase-3 than the parental cells (Figure 5A and B; U). BEZ-235 only slightly increases caspase-3 expression (C, U), while RT increases caspase-3 expression in CDDP-R cells nearly 14-fold (D, U). Predictably, combining RT and BEZ-235 increases caspase-3 expression approximately 7-fold (E, U).

The effect of BEZ-235 on cell proliferation was analyzed via Ki67 staining (Figure 5F-J). CDDP-R cells produce slightly more Ki67 than the parental cells at baseline. BEZ-235 decreases KI67 expression by 58.2%, and RT decreases Ki67 expression in CDDP-R cells by 70.3%.

Combining radiation and BEZ-235 decreases Ki67 expression by 87.8% (Figure 5V). p62 is involved in the transportation of mRNA from the nucleus to the cytoplasm, and thus can also be used to evaluate the level of proliferative activity. CDDP-R cells produce slightly more p62 than the parental cells at baseline (Figure 5K, L, W). BEZ-235 treatment decreases p62 expression by 50%, more than RT (35.4%). Combining radiation and BEZ-235 decreases p62 expression by 74.5%.

Cell growth potential can be assessed by analyzing the development of new vasculature, one marker of which is von Willebrand's factor. Without treatment, CDDP-R cells produce slightly more vWF than the parental cells (Figure 5P, Q). BEZ-235 decreases vWF expression by 75%, whereas RT decreases vWF expression in CDDP-R cells 48%. Combining radiation and BEZ-235 decreases vWF expression 86.1% (Figure 5X). Analysis of several different markers show that BEZ-235 is capable of decreasing the expression of markers of proliferation and growth potential, including Ki67, p62, and von Willebrand factor. Combining BEZ-235 with radiation increases caspase-3, while further decreasing the expression of Ki67, p62, and von Willebrand factor.

## DISCUSSION

After producing a line of cisplatin-resistant cells, we first assessed their resemblance to lung cancer stem cells, a subset of cells thought to play a crucial role in the development of metastatic disease [26]. There is a baseline change in the activation status of the PI3K/Akt/mTOR signaling pathway in the CDDP-resistant cell line (Figure 1). CDDP-R cells show an increase in the phosphorylation of AKT, mTOR, and the S6 ribosomal subunit (Ser 473, 2448, and 240/244, respectively), without a change in total protein expression, indicating that these cells' metabolism mimics the biochemical alterations characteristic of many cancers, and pointing towards a potential therapeutic role for the selective dual pan-class I PI3K and mTOR kinase inhibitor BEZ-235 [19].

We then evaluated whether or not inhibition of this pathway with BEZ-235 would have an effect on cell survival post-CDDP treatment, or on the chemosensitivity of the selected CDDP-R cells. Treatment with BEZ-235 alone did not in and of itself change survival, but combining PI3K/Akt/mTOR inhibition with cisplatin resulted in decreased survival in both the parental and cisplatin-resistant cell lines (Figure 2A). However, ~80% of the cisplatin-resistant cells do survive this combined treatment, whereas only 60% of the parental cells do. Thus, while the PI3K/Akt/mTOR signaling pathway contributes to cisplatin resistance, and blocking it thus restores some of the lost sensitivity, there are clearly other pathways involved in the process as well.

To further explore this effect, we assessed post-treatment phosphorylation of Akt and the ribosomal protein S6. One mechanism by which cell growth is moderated involves altering the processing of mRNA into protein. Successful translation requires, in part, phosphorylation of the S6 protein. Treatment with the drug obliterated the phosphorylation of S6 at Ser 240/244 (Figure 2B), and greatly decreased the phosphorylation of Akt at Ser 473(Figure 2C), in both the parental and CDDP-resistant cell lines. This highlights one potential consequence of blocking the target pathway: pan-proteomal inhibition, resulting in a potential decrease in all protein synthesis.

Little is known about the effects of inhibiting the PI3K/Akt/mTOR signaling pathway on radiosensitivity. As radiation therapy (RT) is a commonly used treatment in many types of cancers, including lung cancer, we next chose to analyze how PI3K/Akt/mTOR inhibition can affect radiosensitivity. Untreated, the cisplatin-resistant cell line shows more resistance to RT than the parental cell line at doses as low as 2Gy (Figure 3). However, PI3K/Akt/mTOR inhibition appears to have a more significant effect on these cells, as survival of the CDDP-resistant cells treated with BEZ-235 is significantly lower than that of comparably treated parental cells (p=0.007). This indicates a great potential for inhibition of this pathway in the treatment of chemoresistant cancers.

Once the effect of radiosensitization was determined, we sought to establish the mechanism by which cell death occurred. We found that the enhanced radiosensitivity of CDDP-R H460 cells was associated with autophagy, but not apoptosis. To assess the induction of autophagy after BEZ-235 treatment and RT, we transfected cells with a GFP-LC3 expression plasmid. LC3 conjugated to phosphatidylethanolamine is present on the surface of autophagosomes. When it is linked to GFP, the formation of autophagosomes can be monitored in real time via fluorescence microscopy. Treatment with either BEZ-235 or radiation increased autophagy in both the parental and radiation-resistant cell lines (Figure 3B). In both cases, BEZ-235 treatment had a greater effect; this was more pronounced in the radiation-resistant cells. Combining the two treatments had an additive effect in the parental cell line. Interestingly, treating the radiation-resistant cells with both BEZ-235 and RT produced a synergistic effect, resulting in a significant increase in measurable levels of autophagy. This again supports the potential utility of inhibiting the PI3K/Akt/mTOR pathway in chemoresistant cancers.

In order to assess the contribution of apoptosis induction to increased post-treatment radio- and chemosensitivity, resistant and parental cells were treated with RT, BEZ-235 + RT, cisplatin, or cisplatin + BEZ-235, and cell lysates assessed for cleaved caspase-3 expression. Analysis of the parental cell line showed that all four of these treatments induced caspase-3 cleavage, though the effect was greater in cells treated with cisplatin or cisplatin + BEZ-235 (Figure 3C) than those exposed to radiation. Thus, in the case of cells derived from the parental line, cisplatin is more effective in inducing apoptosis than is RT. In the CDDP-resistant cell line, cisplatin treatment was able to induce a very minimal amount of apoptosis; RT produced slightly more (Figure 3D). Both amounts are significantly less than that produced by these treatments in the parental cells. Neither combination treatment (BEZ-235 + RT or cisplatin + BEZ-235) resulted in caspase-3 cleavage. Thus, the decrease in cell survival after BEZ-235 treatment is not linked to apoptosis in the CDDP-R cell line, although this may play a role in inhibiting growth of cells from the parental line.

We then moved to an *in vivo* model to assess use of BEZ-235 to treat tumors derived from either the parental or CDDP-resistant cell line athymic nude mice. Tumors were exposed to BEZ-235, irradiation, or both. As expected, combining the treatments resulted in a larger tumor growth delay in tumors derived from CDDP-R cells than either treatment alone (Figure 4). Tissue analysis of animals sacrificed at day 7 showed an increase in caspase-3 production after treatment with radiation, but not BEZ-235; combining the two treatments resulted in an increase midway between the two (Figure 5U). Interestingly, this increase was not seen in the *in vitro* experiment, indicating that apoptosis may play a more significant role *in vivo*.

Ki67 staining revealed a decrease after either treatment alone, which appears to be additive (Figure 5V). Thus, both BEZ-235 and RT decrease proliferation, as expected. These results were confirmed via p62 staining (5W); the decrease noted in p62 expression is indicative of autophagic flux. Finally, we assessed one of the extracellular markers of tumor growth potential: the ability to create a new blood supply. von Willebrand factor, which is expressed on endothelium, served as a marker for potential angiogenesis. Both BEZ-235 and RT decreased von Willebrand factor, as did the combination treatment (Figure 5X), further evidence that cell proliferation is being inhibited.

Overall, we have found that the administration of the selective dual pan-class I PI3K and mTOR kinase inhibitor BEZ-235, in combination with radiation therapy, is a potent addition to the arsenal of tools available to fight cisplatin resistance. The ability of this treatment to significantly lengthen tumor growth delay makes it an attractive prospective treatment for recurrent, drug-resistant cancer.

## MATERIALS AND METHODS

### Cell Culture and Reagents

Parental NCI-H460-Luc2 cells (Caliper Life Sciences, Hopkinton, MA) and CDDP-R H460-Luc2 cells were cultured in RPMI 1640 (American Type Culture Collection, Manassas, VA) supplemented with 10% fetal bovine serum (FBS; Invitrogen, Carlsbad, CA) and 1% penicillin-streptomycin (Invitrogen) at 37°C and humidified 5% CO_2_. Cisplatin (CDDP), basic fibroblast growth factor (bFGF), and insulin were obtained from Sigma-Aldrich (St Louis, MO). Epidermal growth factor (EGF) was obtained from BD Biosciences (San Jose, CA). Methylcellulose medium was purchased from STEMCELL Technologies (Vancouver, BC, Canada). BEZ-235 was obtained from Novartis Pharmaceuticals (Basel, Switzerland).

### Generation of CDDP-Resistant Cells

NCI-H460-Luc2 cells were plated into 100mm × 20mm plates and cultured as described above. After 24 hours, cisplatin was added at a final concentration of 3μM. After incubation for 7 days, the media was removed, the plates washed once with PBS and the cells were trypsinized with 0.05% trypsin-EDTA (GIBCO, Carlsbad, CA). Cells were centrifuged at 4°C for 5 minutes. The pellet was then resuspended in IMDM media (Invitrogen) without serum. After collecting and counting the viable cells via trypan blue staining (0.4% working solution, GIBCO), 1×10^4^ cells were plated in 1% methylcellulose-based (MC-based) serum-free media supplemented with 20ng/mL EGF, 20ng/mL bFGF, and 4μg/mL insulin in ultra-low adherent 24-well cell plates. EGF, bFGF, and insulin were added every 2 days for 14 days. Cells were collected every day for 15 days, washed with PBS twice and gently centrifuged at 4°C for 5 min, and cultured in RPMI 1640 media with 10% FBS.

### Cell proliferation analysis

Parental NCI-H460-Luc2 cells and CDDP-R H460-Luc2 cells were seeded in 96-well plates at a density of 5×10^3^ cells/well. 24h later, DMSO, CDDP (3μM), BEZ-235 (50nM) or CDDP+BEZ-235 was added to each well for 72 h. Cell proliferation assay was assessed using the CellTiter 96® AQ_ueous_ Non-Radioactive Cell Proliferation Assay (Promega, Madison, WI). As per kit instructions, 20 μL of the combined MTS/PMS solution was added into each well containing 100μL of cells in culture media. The plate was incubated for 1–4 h at 37 °C in a humidified 5% CO_2_ atmosphere. Absorbance was measured at 570nm with a Flexstation 3 Molecular Device (Sunnyvale, CA).

### Immunoblotting

Cells were washed twice with ice-cold PBS before the addition of lysis buffer (m-PER Mammalian Protein Extraction reagent, Thermo Scientific, Waltham, MA) and inhibitor cocktail (Sigma, 5μg/mL). Protein concentration was quantified by the Bio-Rad method. 35μg of protein were loaded into each well of a 12.5% SDS-PAGE gel, followed by transfer onto PVDF-membranes (Bio-Rad, Hercules, CA). Membranes were blocked with 5% non-fat dry milk in PBS-T for 1 hour at room temperature. The blots were then incubated with antibodies against the following human proteins: p-mTOR (1:1000; Cell Signaling Technology, Danvers, MA); mTOR (1:1000; Cell Signaling Technology); p-Akt (1:1000; Cell Signaling Technology, Danvers, MA); Akt (1:1000; Cell Signaling Technology); p-S6 ribosomal protein (1:1000; Cell Signaling Technology); S6 ribosomal protein (1:1000; Cell Signaling Technology); caspase-3 (1:1000; Cell Signaling Technology); and actin (1:1000; Cell Signaling Technology) for 1 hour at 4°C. Blots were rinsed 3×10min, then incubated with goat anti-rabbit IgG secondary (1:5000; Santa Cruz Biotechnology, Santa Cruz, CA) for 45 minutes at room temperature. Immunoblots were developed by using the chemiluminescence detection system (PerkinElmer, Waltham, MA), according to the manufacture's protocol, and autoradiography.

### *In Vitro* Clonogenic Assays

Parental NCI-H460-Luc2 cells and CDDP-R H460-Luc2 cells were treated with DMSO or BEZ-235 (50nM). After incubation for 24 hours, the cells were irradiated with 0-6Gy using a ^137^Cs irradiator (J.L. Shepherd and Associates, Glendale, CA) at room temperature (dose rate 1.8 Gy/min). Cells were washed twice with PBS, then incubated at 37°C for 8 to 10 days. Cells were then fixed for 15 minutes with 3:1 methanol: acetic acid and stained for 15 minutes with 0.5% crystal violet (Sigma) in methanol. After staining, colonies were counted using a cutoff of 50 viable cells. Surviving fraction was calculated as (mean colony counts)/(cells inoculated) × (plating efficiency), where plating efficiency was defined as (mean colony counts)/(cells inoculated for nonirradiated controls). The radiation dose enhancement ratio (DER) was calculated as the dose (Gy) of radiation plus vehicle (DMSO) divided by the dose (Gy) of radiation plus BEZ-235 (normalized for BEZ-235 toxicity) necessary for a surviving fraction of 0.25. Experiments were conducted in triplicate and mean, SD, and p values were calculated.

### Autophagy Assay

2×10^5^ parental NCI-H460-Luc2 cells or CDDP-R H460-Luc2 cells were transfected with 2.5μg of GFP-LC3 expression plasmid (gift from Dr. Norboru Mizushima) using Lipofectamine 2000 (Invitrogen). After 24 hours, cells were treated with BEZ-235 (50nm) for 2 hours followed by treatment with 5Gy radiation. After 48 hours, the fluorescence of GFP-LC3 was observed using confocal fluoroscopy.

### Development of xenografts, treatment, and assessment of tumor growth

A suspension of 1×10^6^ NCI-H460-Luc2 cells or CDDP-R H460-Luc2 cells in 50 mL PBS was injected subcutaneously into the left posterior flank of female athymic nude mice (nu/nu, 5–6 weeks old; Harlan Sprague Dawley Inc., Indianapolis, IN). Tumors were allowed to grow for 6–8d until average tumor volume reached 0.1cm^3^. Mice were divided into the following treatment groups (5 mice per group): Parental cells + vehicle; CDDP-resistant cells + vehicle; CDDP-resistant cells + BEZ-235; CDDP-resistant cells + RT; CDDP-resistant cells + BEZ-235 + RT. BEZ-235 was formulated in N-methyl-2-pyrrolidone (NMP)/PEG300 (1/9, v/v). NMP/PEG300 or BEZ-235 in NMP/PEG300 was given daily by intraperitoneal (i.p.) injection at doses of 35mg/kg for 7 consecutive days. In the case of combination treatment, drug or vehicle was given for 2d prior to the first dose of irradiation. Mice in radiation groups were irradiated 1h after drug or vehicle treatment with daily 2Gy fractions given over 5 consecutive days. Tumors on the flanks of the mice were irradiated using an X-ray irradiator (Therapax, Agfa NDT, Inc., Lewis Town, PA). Tumors were measured 2–3 times weekly in 3 perpendicular dimensions using a Vernier caliper. Volume was calculated using the modified ellipse volume formula (volume = (height*width*depth)/2). Growth delay was calculated for treatment groups relative to control tumors.

### Immunohistochemical analysis

Mice were implanted with parental NCI-H460-Luc2 cells or CDDP-R H460-Luc2 cells and treated as described above. After 7d of daily treatments, mice were sacrificed and tumors were paraffin fixed. Slides from each treatment group were then stained for von Willebrand factor (vWF) using anti-vWF polyclonal antibody (1:100, Chemicon, Billerica, MA). Blood vessels were quantified by randomly selecting 400X fields and counting the number of blood vessels per field. This was done in triplicate and the average of the three counts was calculated. Ki67, active caspase-3 and P62 staining were performed in the Vanderbilt University pathology core laboratory using standard protocols. Number of positive cells per field were scored and graphed by averaging three repeated assessments.
